# Antiviral activity of flavonoids present in aerial parts of *Marcetia taxifolia* against Hepatitis B virus, Poliovirus, and Herpes Simplex Virus *in vitro*

**DOI:** 10.17179/excli2019-1837

**Published:** 2019-11-05

**Authors:** Joseph Thomas Ortega, María Luisa Serrano, Alírica Isabel Suárez, Jani Baptista, Flor Helene Pujol, Lucía Vicenta Cavallaro, Héctor Rodolfo Campos, Héctor Rafael Rangel

**Affiliations:** 1 Laboratorio de Virología Molecular, Centro de Microbiología y Biología Celular, Instituto Venezolano de Investigaciones Científicas, Caracas, Venezuela; 2 Cátedra de Virología, Facultad de Farmacia y Bioquímica, Universidad de Buenos Aires, Argentina; 3 Unidad de Química Medicinal, Facultad de Farmacia, Universidad Central de Venezuela, Caracas, Venezuela; 4 Laboratorio de Productos Naturales, Facultad de Farmacia, Universidad Central de Venezuela, Caracas, Venezuela

**Keywords:** antiviral, flavonoids, Marcetia taxifolia, HBV, HSV, polio

## Abstract

*Marcetia taxifolia* is a neotropical plant present in South America and it has been evaluated in several biological models due to the presence of active metabolites. Nevertheless, there is a limited quantity of studies related to the antiviral activity of the compounds present in this *genus*. In our work, the antiviral effect of the compounds isolated from the aerial parts of *Marcetia taxifolia *was evaluated against Hepatitis B virus (HBV), Herpes Simplex Virus type 1 (HSV-1), and Poliovirus type 1 (PV-1). The cytopathic effect and viral quantification by qPCR were determined as indicative of antiviral activity. Our data show that myricetin rhamnoside (MyrG), myricetin-3-α-O-ramnosil (1→6)-α-galactoside (MyrGG), 5,3'-dihydroxy-3,6,7,8,4'-pentamethoxyflavone (PMF), 5-hydroxy-3,6,7,3',4'pentamethoxyflavone (PMF-OH) had antiviral activity without cytotoxic effects. The methoxyflavones PMF and PMF-OH were the most active compounds, showing an antiviral effect against all the evaluated viruses. Computational studies showed that these compounds could interact with the Reverse Transcriptase. Altogether, these results suggest that the flavonoids (related to myricetin and methoxyflavones) are the main antiviral compounds present in the aerial parts of *Marcetia taxifolia*. Furthermore, our results showed that the methoxyflavones have a broad antiviral activity, which represents an opportunity to evaluate these flavonoids as lead molecules to develop new antiviral compounds.

## Introduction

Emergence, resistance and spreading of viral strains promote research for new antiviral drugs. In fact, several biological compounds are found in plants and some of these plant extracts have shown broad antiviral properties. These inhibitory effects could be related to blocking main viral enzymes directly or in some cases, to be modulating a cellular pathway (Arena et al., 2004[1]; Jassim and Naji, 2003[16]; Sharma et al., 2009[40]). These molecular mechanisms are similar to the drugs used in antiviral therapy: direct-acting antiviral and drugs that block a host cellular process avoiding viral replication (Ortega et al., 2013[32]).

*Marcetia* is a Neotropical *genus* distributed from Venezuela to Uruguay (Berry and Luckana, 2001[4]). Baptista et al. (2016[3]) described for the first time the compounds present in the aerial parts of *Marcetia taxifolia*. These compounds are: marcetol (1), 5-α-cholestan-3-β-ol (2), 5-α-cholestan-7-en3-β-ol (3), 24,25-dehydropollinastanol (4), lupa-12,20(29)-dien-3-ol (5), 5,3'- dihydroxy-3,6,7,8,4'-pentamethoxyflavone (6), 5-hydroxy-3,6,7,3',4'pentamethoxyflavone (7), myricetrine (8) and myricetin-3-α-O-ramnosil (1→6)-α-galactoside (9). Previous reports have shown that these methoxyflavones and myricetrine have antiviral activity against HIV-1 (Ortega et al., 2017[34][33]). Additionally, Leite et al. have reported the antibacterial and antifungal activities of *Marcetia taxifolia* extract. These activities are principally associated with polymethoxylated flavonoids (Leite et al., 2012[21]). However, the antiviral activity of these compounds has been scarcely evaluated. Thus, in this study the compounds obtained from the aerial parts of *Marcetia taxifolia *were evaluated against three different viral families of public health concern: Hepatitis B virus (HBV), Herpes simplex virus (HSV) and Poliovirus (PV). HBV affects around 240 million people worldwide and is related to hepatic cirrhosis and hepatocellular carcinoma (WHO, 2019[46]). HSV may produce oral or genital lesion depending on the virus type (HSV-1 and HSV-2 respectively) (Whitley and Baines, 2018[45]) and Poliovirus is associated with paralysis in unvaccinated children (Menant and Gandevia, 2018[26]). These viruses exhibit many differences in the replicative cycle. Therefore, the aim of this study was to evaluate the antiviral activity of compounds isolated from *Marcetia taxifolia* against these different viral families.

## Material and Methods

### Cell culture, viruses, and compounds

HepG2.2.15 cells were kindly donated by Dr. Isabelle Chemin (Team leader Hepatocarcinogenesis and viral infections) from INSERM, France. These cells were grown in RPMI medium supplemented with 10 % fetal bovine serum (Gibco), 100 IU/mL penicillin G, 100 µg/mL streptomycin, 0.25 µg/mL amphotericin B and 200 µg/mL G418 and Vero cells in Eagle medium supplemented with 10 % fetal bovine serum (Gibco), 100 IU/ml penicillin G, 100 µg/mL streptomycin at 37 °C under 5 % CO_2_ in a CO_2_ incubator. Herpes simplex type 1 F strain (HSV-1), poliovirus type 1 Sabin strain (PV-1) of viral repository stocks from “Cátedra Virología, Facultad de Farmacia y Bioquímica, UBA, Argentina”.

The *Marcetia taxifolia* (A.St.-Hil.) DC., plant collection, verification, and compound isolation were previously described (Baptista et al., 2016[3]). The *Marcetia taxifolia* (A.St.-Hil.) DC. (*Melastomataceae*) was collected in the Amazonanian state in Venezuela. The chemical constituents were separated through organic extraction, further isolation was developed by chromatographic techniques and the compounds were characterized using spectroscopic and spectrometric methods.

### Cytotoxicity assay

The cytotoxicity assays were determined by the MTT method (Mosmann, 1983[28]). Also, the cells were seeded in 96-wells plate at a density of 1.5 x 10^4^ cells/well. The next day, different concentrations of the flavonoid compounds were added to the cells and 24 h later, the cell viability was evaluated using the MTT (ThermoFisher, USA) cell proliferation assay as previously described (Ortega et al., 2017[34]). 

### HBV antiviral assay

HepG2.2.15 constitutively produces HBV particles and is broadly used to evaluate the antiviral activity (Sells et al., 1988[39]). The HBV production was quantified through a real-time PCR methodology, using as target the X gene. The sequence of specific primers was forwarded-1608-1627: ATG GAG ACC ACC GTG AAC GC and reversed-1887-1868 AGG CAC AGC TTG GTG GCT TG. The amplification product was of approximately 260 bp. Briefly, 24 h prior to the antiviral assay, the cells were seeded at a density of 1.2 x 10^4^ cells / well for each condition. The cells were incubated with “Infection medium” (RPMI with 2 % FBS and 1 % DMSO) (Quintero et al., 2011[38]) with and without the compounds at the described concentrations. After 72 h, the medium and the compounds were refreshed by replacing them and incubated by 72 h. The supernatant was collected and viral DNA extracted using High Pure Viral Nucleic Acid Kit (Roche Life Science, Germany). The development of qPCR was done on Applied Biosystems 7300 by using a Sybr green commercial kit (Luna® Universal qPCR, Kit NEB Co. USA). The amplification conditions were the following: 3 min at 95 °C, 40 cycles of 15 sec. at 95 °C, 30 sec. at 61 °C and 30 sec. at 72 °C. The viral inhibition relative to control (%) was calculated. Statistical analyses of at least three independent experiments were performed. 

### Herpes virus and polio antiviral assay

To determine the antiviral activity against Herpes and Polio, a plaque reduction assay, on 24 well culture plates was performed. The Vero cells were infected with 100 plaque forming units (PFU) of HSV-1 or PV-1. After 60 min adsorption at 37 °C and 5 % CO_2_ the virus was removed. Then, the monolayers were washed twice with PBS and overlaid with plaque medium (0.5 % Methylcellulose). Next, the compounds were added at different concentrations in each well and incubated by 24 or 48 h for PV-1 and HSV-1 respectively. Finally, cells were fixed with 10 % formalin and stained with 0.4 % crystal violet. Viral infection, cellular morphology, and cytotoxic controls in each assay were all performed. 

### 3D model building and refinement

#### Homology modeling

A homology model of the Hepatitis B virus Reverse Transcriptase (HBV-RT) was generated by using the crystallographic structure of HIV-1 Reverse Transcriptase (HIV-1-RT) (PDB 3V81) as the template (Daga et al., 2010[8]; Das et al., 2012[9]). The initial model was obtained with the SWISS-MODEL modeling server (Arnold et al., 2006[2]) and the tools of DeepView/Swiss-PdbViewer 4.01 software (Guex and Peitsch, 1997[13]). In this model, hydrogen atoms were added, and the partial charges were assigned for energy refinement. After that, the protein model was embedded in a 100 Å water box. Then, energy minimization preserving the enzyme folding and optimizing the relative position between the water molecules and the fixed backbone atoms was performed. The obtained system underwent a one nanosecond (ns) molecular dynamic (MD) simulation with the following characteristics: (a) Periodic Boundary Conditions (PBC) were introduced to stabilize the simulation space; (b) the long-range electrostatic potential was treated by the Particle Mesh Ewald summation method (PME) (Toukmaji et al., 2000[42]); (c) Newton's equation using the r-RESPA method (Masella, 2006[25]) (every 4 femtoseconds (fs) for long-range electrostatic forces, 2 fs for short-range non-bonded forces, and 1 fs for bonded forces) was integrated; (d) the temperature was maintained 300 ± 5 K by means of Langevin's algorithm (Izaguirre et al., 2012[15]); (e) Lennard-Jones (L-J) interactions were calculated with a cut-off of 14 Å, the switching distance was 10 Å, and the non-bonded pair list distance was 14 Å; (f) a frame every 5 pico seconds (ps) was stored, yielding 2000 frames; (g) constraints with a force constant of 1000kcal/mol/Å^2 ^were applied to the backbone atoms (N, Ca, C, O) and gradually decreased during the simulation. The simulations were carried out in three phases: an initial period of heating from zero to 300 K over nine ps; an equilibration MD simulation of 0.1 ns, and monitored MD phase of simulation of one ns. The lowest energy frame of the MD simulation was further minimized and represented in the final structure. MD simulations described in the study were performed with NAMD 2.8 (Phillips et al., 2005[37])/Vega ZZ 3.1.0.21, using the CHARMM force field (Vanommeslaeghe et al., 2010[44]) and the Gasteiger charges. All reported minimizations were performed using the conjugate gradient algorithm until a root-median square (rms) gradient was smaller than 0.01 kcal/mol/Å. The final model validation was carried out with ProSA (Wiederstein and Sippl, 2007[47]) and PROCHECK programs (Laskowski et al., 1993[20]).

### Molecular docking with HBV-RT

The 3D structure of each inhibitor was obtained from the PubChem database (Kim et al., 2016[18]) or generated by parent structure modification with VegaZZ 3.1.0.21 (Pedretti et al., 2004[36]). The molecular docking was performed with AutoDock 4/VegaZZ 3.1.0.21 and 30 runs conducted for each compound. Sampling in a cube of 10 Å sides around the ligand was carried out. The results were prioritized according to the predicted free energy of binding. Then the final complexes, keeping the backbone atoms of the protein fixed and improving the mutual adaptability between ligand and enzyme were minimized.

## Results

The antiviral activity of the compounds isolated from the aerial parts of *M. taxifolia* in *in vitro* models of HBV, HSV-1, and PV-1 was assayed. Table 1(Tab. 1) summarizes the results for cytotoxicity and viral inhibition for the evaluated compounds. As indicated in this table, only: myricetin rhamnoside (MyrG), myricetine-3α-O-rhamnosyl (1→6)-α-galactoside (MyrGG), 5,3'-dihydroxy-3,6,7,8,4'-pentamethoxyflavone (PMF) and 5-hydroxy-3,6,7,3',4'-pentamethoxyflavone (PMF-OH), were active against at least one of the evaluated viruses. Additionally, these compounds showed a scarce or non-cytotoxic effect.

### Antiviral activity on HSV-1 and PV-1 models

**Figure 1:**
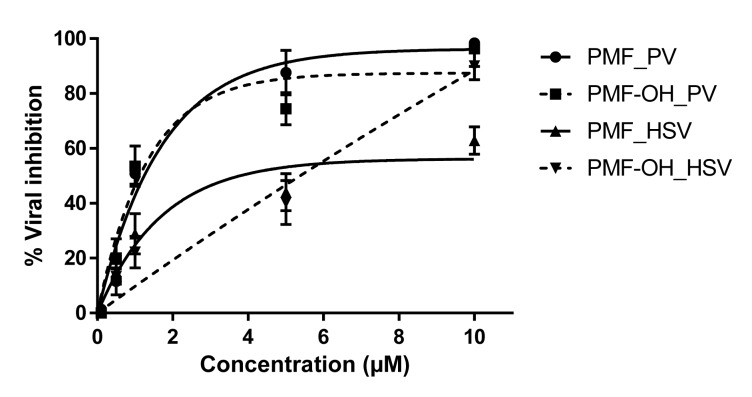
Antiviral activity of methoxyflavones against PV-1 and HSV-1

The antiviral activity of each compound against Polio-1 and HSV-1 was assayed. The myricetin related compounds did not show antiviral activity against these viruses. However, methoxyflavones exhibited activity with an EC_50_ near to 1 µM against PV-1 (Figure 1(Fig. 1)). An inhibitory effect of 90 % (PMF) and 60 % (PMF-OH) against HSV-1 was observed at a concentration of 10 μM (Figure 1(Fig. 1)).

### Antiviral activity on the HBV model

**Figure 2:**
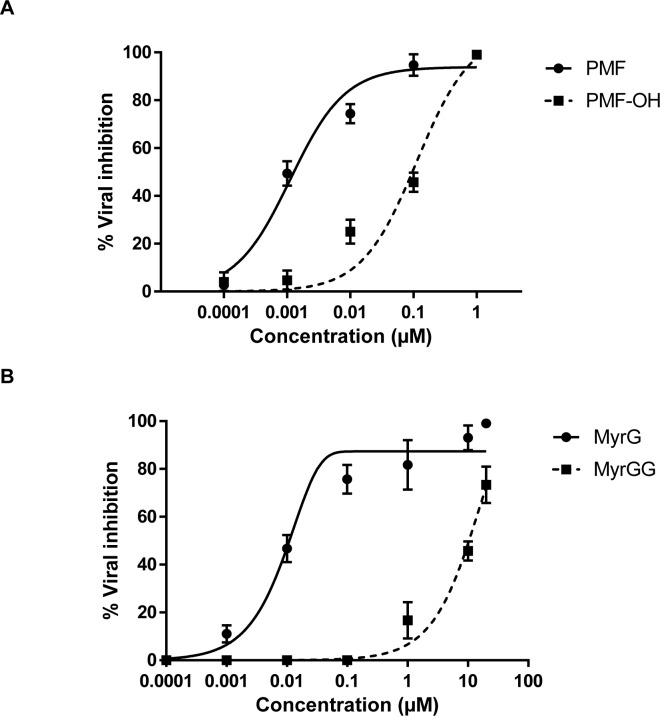
Antiviral activity of *Marcetia taxifolia* flavonoids against HBV. (A) methoxyflavones: PMF and PMF-OH, (B) Myricetin glycosides: MyrG and MyrGG

The selectivity index (SI) of the methoxyflavones (PMF and PMF-OH) against HBV was 10,000 and 102 respectively, with EC_50_ values of 0.001 µM for PMF and 0.098 µM for PMF-OH. These compounds showed the highest antiviral activity against HBV (Figure 2A(Fig. 2)). Additionally, MyrG presents an SI of 6667 and an EC_50_= 0.015 µM while MyrGG showed an SI of 8.3 and EC_50_= 12 µM (Figure 2B(Fig. 2)).

Previous reports show that glycosylated flavonoids related to myricetin, as well as methoxyflavones, can inhibit the HIV-1-RT (Ortega et al., 2017[33]). Thus, the interaction of these compounds with the HBV reverse transcriptase (HBV-RT) as a potential target was evaluated *in silico*. To date, there is no experimental crystal structure of HBV-RT available. For this reason, a homology model of the HBV-RT was constructed by using the crystal structure of HIV-1-RT as a template.

### Homology modeling

**Figure 3:**
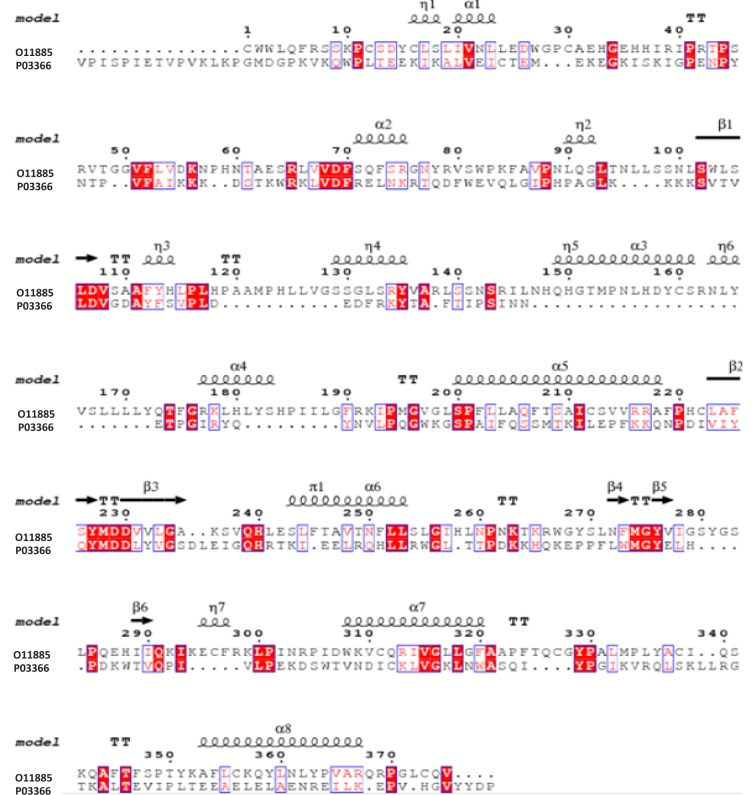
Sequence alignment of HBV-RT and HIV-1-RT polymerases. Secondary structure prediction of HBV-RT. Predicted amino acid sequences of HBV-RT (O11885) and HIV-1-RT PDB code 3v81 (P03366) were accordingly to Daga et al. (2010) conserved residues, which are highlighted with a red background.

The alignment between HBV-RT and HIV-1-RT (UniProtKB O11885 and P03366) was performed using the structural alignment tool of Swiss-PdbViewer/DeepView (Figure 3(Fig. 3)). The HIV-1-RT and HBV-RT share many of the residues involved in key protein-ligand interactions (Daga et al., 2010[8]). These include the YMDD motif and the catalytic triad of aspartic acid residues, Asp107, Asp229, Asp230. Some differences in the putative hydrophobic NNRTI-binding pocket were found. However, the insertion of 4 amino acids in the HBV enzyme was the major difference observed. Altogether, data suggest that HIV-1-RT and HBV-RT share enough structural and functional similarity to serve as a putative predictor for the interaction with RT inhibitors.

**Figure 4:**
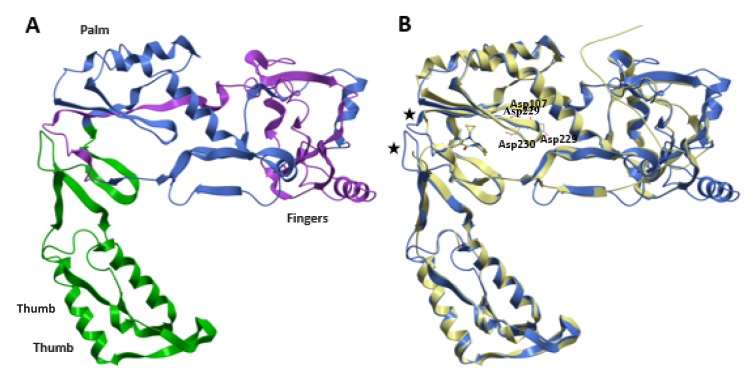
Comparison between the homology model of HBV-RT and the crystal structure of HIV-1-RT. (A) Ribbon diagram of HBV-RT. Three subdomains are described: Fingers (residues 1-49 and 90-172, purple), palm (residues 50-89 and 173-267, blue), and thumb (residues 268-351, green). (B) HBV-RT (blue) and HIV-1-RT (yellow) are superimposed; regions showing significant differences are marked (*). Amino acids of the catalytic triad and Nevirapine are displayed in sticks.

A structural model of the HBV-RT using the SWISS-MODEL package was generated (Figure 4(Fig. 4)). Three main subdomains in the HBV-RT structure were identified: the finger, residues 1-49 and 90-172, the palm, residues 50-89 and 173-267 and the thumb, residues 268-351. Nevertheless, differences between the models were found (Figure 4(Fig. 4)). Homology comparison between HBV-RT model and HIV-1-RT template suggested a similar structural conformation with a root mean square deviation (rmsd) of 2.1 Å for the Cα atoms (Table 2(Tab. 2)). 

The conformational differences related to the secondary structure between HIV-RT and HBV-RT were determined. The main HBV features were the elongations in 4 and 5 residues occurring in the protein loops (Figure 4 B(Fig. 4)). The palm contains the polymerase active site and the non-nucleoside pocket located ~10 Å apart. No differences were observed around the three aspartate residues of the catalytic triad: Asp107, Asp229, and Asp230, in HBV and corresponding in HIV-1 RT to Asp110, Asp185, and Asp186.

### Docking at the NNRTI binding pocket

**Figure 5:**
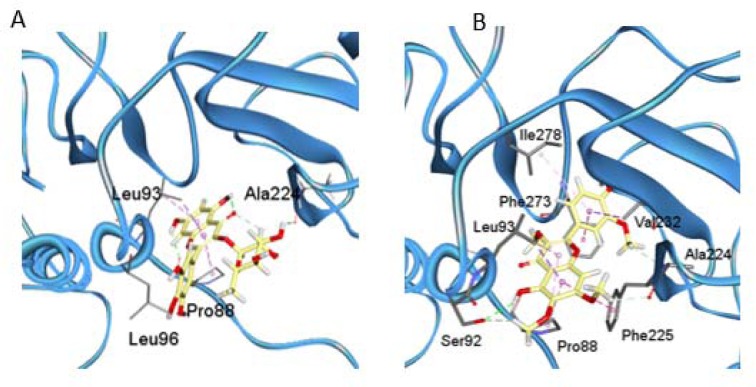
Docking results of MyrG (A) and PMF (B) over the HBV-RT model. 3D representations of the complex interactions categorized by hydrogen bonds (green dashed lines), π-stacks (pink-dashed lines).

Some flavonoids have been characterized as HIV-1-RT inhibitors. Furthermore, previous reports showed that these compounds, particularly myricetin and myricetin 3-rhamnoside, could occupy the NNRTI pocket (Li et al., 2011[22]; Ortega et al., 2017[34]). Thus, molecular docking study of glycosylated flavonoids (MyrG and MyrGG) and methoxyflavones (PMF and PMF-OH) into the NNRTI pocket on the HBV-RT model were carried out. Figure 5(Fig. 5) shows the docking representation of the MyrG (A) and PMF (B) on the HBV-RT model, and Table 3(Tab. 3) summarizes the docking scores for the ligands evaluated in this study. The compounds that best interact with the NNRTI pocket were the methoxyflavones. However, the best-docked compound was PMF-OH, with a binding energy of -5.59 kcal/mol. The compound PMF shows binding energy of -5.33 kcal/mol. However, the glycosylated flavonoids showed lower binding energy than methoxyflavones. The best ranked binding energy was for MyrG with a binding energy of -4.61 Kcal/mol. All these results are summarized in Table 3(Tab. 3).

For more results see the Supplementary material.

## Discussion

Plant-derived compounds represent a broad variety of chemical families. However, flavonoids have been the most widely studied as possible antivirals. Indeed, some of these compounds have been active against viruses such as HBV, Polio, and Herpes virus (Zhang et al., 2014[49]; Eggers, 1985[11]; Parvez et al., 2016[35]; Huang et al., 2017[14]). *Marcetia genus *plants are a source of flavonoids and have been barely studied. Thus, to determine the antiviral activity of the nine compounds isolated from the aerial parts of *M. taxifolia*, we selected three different *in vitro* models: HBV, HSV-1, and PV-1. 

The results showed that only the glycosylated flavonoids related to myricetin and the methoxyflavones exhibited antiviral activity. Myricetin related compounds and methoxyflavones were active against HBV (Figure 2(Fig. 2)). However, the most potent compounds were the methoxyflavones (Figure 2A(Fig. 2)). In our best knowledge, this is the first report that indicates the antiviral activity of these compounds on HBV. Interestingly, other flavonoids related to myricetin such as quercetin have exhibited an antiviral effect against HBV (Cheng et al., 2015[7]). The methoxyflavones have been barely studied and there are just a few reports related to their antiviral activity. (Ortega et al., 2017[34]; Nagai et al., 1995[29]; Eggers, 1985[11]; De Meyer et al., 1991[10]). 

Flavonoids and related compounds can interact with polymerases, especially with the reverse transcriptase (Kuete et al., 2010[19]; Ono et al., 1990[31]). Ortega et al. (2017[34][33]) described that myricetin and methoxyflavones may act as non-nucleoside Reverse Transcriptase inhibitors (NNRTI). Indeed, a Reverse Transcriptase is involved in the HBV replication cycle and this enzyme has a high similarity with the HIV-1-RT. The binding of MyrG and MyrGG on the reported NNRTI binding pocket was shown by molecular docking. Moreover, the binding energy was inversely proportional to glycosylation grade (Figure 5(Fig. 5) and Table 3(Tab. 3)). The methoxyflavones showed lower binding energies compared to the glycosylated compounds. Additionally, these compounds produce more hydrogen bonds, which suggest a more stable interaction with the HBV-RT. These docking results are in concordance with the antiviral activities obtained in the *in vitro* model of HBV. Residues that consistently within 5 Å from docked ligands were Val87, Pro88, Asn89, Leu90, Ser92, Leu 93, Trp 103, Leu223, Ala224, Phe225, Val232, and Phe273. As shown, these results suggest the hydrophobic NNRTI pocket of HBV-RT as a potential target for new inhibitors. 

To determine the antiviral spectrum of these flavonoids, two additionally viral species were selected as models. PV-1 (a single strain RNA virus) and HSV-1 (a DNA virus), which diverged from HBV in the replication mechanisms. Our data show that the glycosylated flavonoids did not have an inhibitory effect against Polio nor Herpes. These observations are in concordance with previous reports for these myricetin related compounds (Lin et al., 2000[23]; Castrillo et al., 1986[6]). The methoxyflavones displayed a potent effect against Polio and these results are in agreement with previous reports of similar compounds versus this virus (Van Hoof et al., 1984[43]; De Meyer et al., 1991[10]). However, the effect of the methoxyflavones against HSV-1 (Figure 1(Fig. 1)) was modest compared to those observed against HBV (Figure 2a(Fig. 2)). Altogether, these results showed that the methoxyflavones are active against Polio, Herpes, and Hepatitis B. Additionally, as previously mentioned the methoxyflavones can inhibit influenza virus and HIV-1. These viruses do not share any replicative step thus; the broad range of antiviral activity of methoxyflavones may suggest two possible inhibitory mechanisms: a) inhibiting viral polymerases by acting as non-selective inhibitors, or b) modulating a cellular process related to the viral replication cycle. Indeed, methoxyflavones have been described as inhibitors of several cellular processes, such as the cell cycle, apoptosis, kinases pathway, NFKb/AKT pathway and others (Wudtiwai et al., 2011[48]; Bhardwaj et al., 2016[5]; Faqueti et al., 2016[12]; Kim et al., 2014[17]; Ma et al., 2015[24]; Merlo et al., 2015[27]; Shi et al., 2013[41]; Nakao et al., 2011[30]). 

In conclusion, the methoxyflavones showed antiviral activity against all the evaluated viruses, while the glycosylated flavonoids related to myricetin were active only on HBV. In the HBV model, the possible antiviral mechanism could be related to HBV-RT inhibition. On the other hand, the results obtained for PMF against HSV-1 and PV-1 suggest that these compounds could have another mechanism related to a cellular process modulation. However, additional pharmacological studies to establish the target of the methoxyflavones and further evaluations of glycosylated flavonoids as RT inhibitors are needed. Altogether, these results show the potential of flavonoids as antiviral lead compounds. 

## Notes

Héctor Rodolfo Campos and Héctor Rafael Rangel (Laboratorio de Virología Molecular, Centro de Microbiología y Biología Celular, Instituto Venezolano de Investigaciones Científicas, Caracas, Venezuela; Tel: 58 212 5041874, E-mail: hrangel@ivic.gob.ve, hrangel2006@gmail.com) contributed equally as corresponding authors.

## Acknowledgements

This work was supported by the Agencia Nacional de Promoción Científica y Tecnológica (ANPCyT; PICT2014-1672) to RC at Cátedra de Virología, Facultad de Farmacia y Bioquímica, Universidad de Buenos Aires, IVIC project number 495. To HR at Laboratorio de Virología Molecular, Caracas, Venezuela, and UNU Biotechnology Program for Latin America and the Caribbean UNU-BIOLAC to OJT, FP and RC.

## Supplementary Material

Supplementary material

## Figures and Tables

**Table 1:**
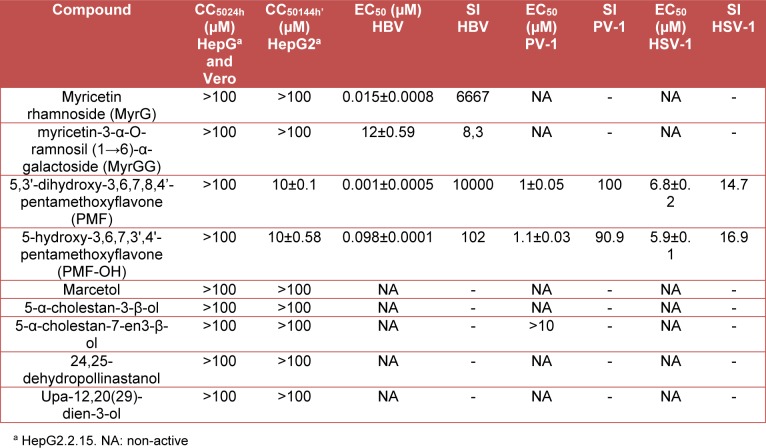
Cytotoxic concentration and Inhibitory concentration of the tested compounds

**Table 2:**
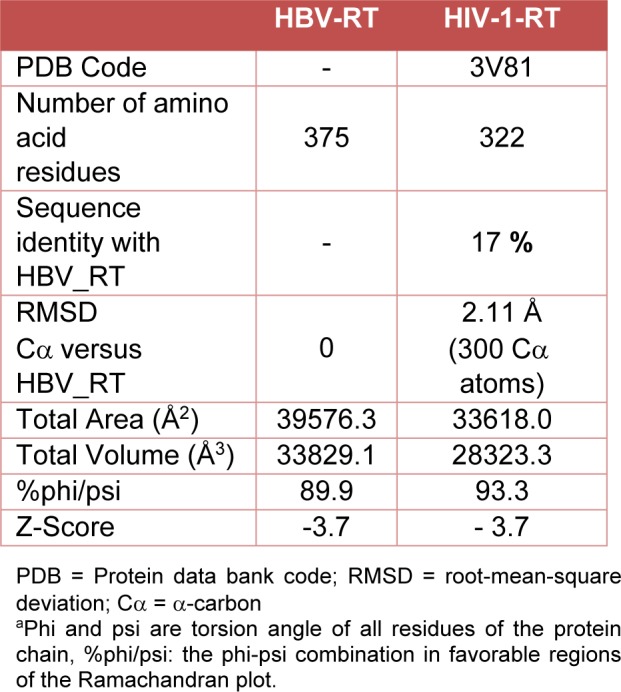
Structural comparison between the HBV-RT model and HIV-1-RT

**Table 3:**
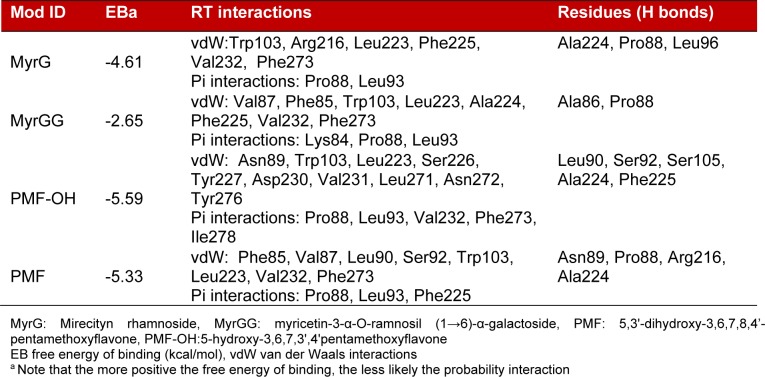
Docking results of the evaluated compounds using the HBV-RT model
