# Supplementary material for: Antiviral activity of flavonoids present in aerial parts of Marcetia taxifolia against Hepatitis B virus, Poliovirus, and Herpes Simplex Virus in vitro
Source: EXCLI J. 2019 Nov 5;18:1037–48. doi: 10.17179/excli2019-1837 (PMC6868923; doi:10.17179/excli2019-1837)
Supplement: Supplementary material [file EXCLI-18-1037-s-001.pdf]

EXCLI Journal 2019;18:1037-1048 – ISSN 1611-2156
Received: September 20, 2019, accepted: October 29, 2019, published: November 05, 2019

Supplementary material to:

ANTIVIRAL ACTIVITY OF FLAVONOIDS PRESENT IN AERIAL PARTS OF *MARCETIA TAXIFOLIA* AGAINST HEPATITIS B VIRUS, POLIOVIRUS, AND HERPES SIMPLEX VIRUS *IN VITRO*

Joseph Thomas Ortega[1,2], María Luisa Serrano[3], Alírica Isabel Suárez[4], Jani Baptista[4], Flor Helene Pujol[1], Lucía Vicenta Cavallaro[2], Héctor Rodolfo Campos[2*], Héctor Rafael Rangel[1*]

1 Laboratorio de Virología Molecular, Centro de Microbiología y Biología Celular, Instituto Venezolano de Investigaciones Científicas, Caracas, Venezuela
2 Cátedra de Virología, Facultad de Farmacia y Bioquímica, Universidad de Buenos Aires, Argentina
3 Unidad de Química Medicinal, Facultad de Farmacia, Universidad Central de Venezuela, Caracas, Venezuela
4 Laboratorio de Productos Naturales, Facultad de Farmacia, Universidad Central de Venezuela, Caracas, Venezuela

* Corresponding authors: Héctor Rodolfo Campos: Cátedra de Virología, Facultad de Farmacia y Bioquímica, Universidad de Buenos Aires. E-mail: rcampos@ffyb.uba.ar; Héctor Rafael Rangel: Laboratorio de Virología Molecular, Centro de Microbiología y Biología Celular, Instituto Venezolano de Investigaciones Científicas, Caracas, Venezuela. Tel: 58 212 5041874, E-mail: hrangel@ivic.gob.ve ; hrangel2006@gmail.com

http://dx.doi.org/10.17179/excli2019-1837

This is an Open Access article distributed under the terms of the Creative Commons Attribution License (http://creativecommons.org/licenses/by/4.0/).

EXCLI Journal 2019;18:1037-1048 – ISSN 1611-2156
Received: September 20, 2019, accepted: October 29, 2019, published: November 05, 2019

Supplementary Table 1: Antiviral activity of 5,3'-dihydroxy-3,6,7,8,4’-pentamethoxyflavone (PMF) and 5-hydroxy-3,6,7,3',4'pentamethoxyflavone (PMF-OH) against Polio (**a**) and Herpes simplex virus (**b**) *in vitro*. Plaque forming unit values for Poliovirus and HSV on Vero cells. Plaque forming units (PFU) were determined by crystal violet staining as described in ’Material and Methods’ section. Values are shown as PFU value for 3 independent experiments for each condition. PFU values obtained for each virus as control are shown.

a)	Polio virus											
**Concentration (µM)**	PMF (PFU)			PMF-OH (PFU)			PMF			PMF-OH		
							Median (%)*	SD	N	Median (%)*	SD	N
**0.0001**	91	92	89	92	95	90	0.1	1.39	3	0.1	2.52	3
**0.001**	81	80	78	76	70	73	11.48	1.25	3	18.89	2.50	3
**0.01**	45	41	48	42	40	48	50.37	3.19	3	51.85	4.16	3
**0.1**	11	16	10	23	20	26	86.30	2.92	3	74.44	3.00	3
**1**	1	1	4	3	5	2	97.78	1.57	3	96.30	1.53	3

PFU for Polio virus control: 92 and SD 3
*Inhibition percent (%) relative to control

b)	Herpes simplex virus											
**Concentration (µM)**	PMF (PFU)			PMF-OH (PFU)			PMF			PMF-OH		
							Median (%)*	SD	N	Median (%)*	SD	N
**0.0001**	98	91	90	96	98	90	3.14	3.71	3	1.40	3.54	3
**0.001**	82	68	76	80	82	86	21.53	5.97	3	13.90	2.59	3
**0.01**	68	73	61	76	73	70	29.86	5.13	3	23.96	2.55	3
**0.1**	48	58	55	67	55	50	44.10	4.36	3	40.28	7.43	3
**1**	34	30	37	10	5	10	64.93	2.99	3	91.32	2.45	3

PFU for HSV control: 96 and SD 5
*Inhibition percent (%) relative to control

EXCLI Journal 2019;18:1037-1048 – ISSN 1611-2156
Received: September 20, 2019, accepted: October 29, 2019, published: November 05, 2019

Supplementary Table 2: Antiviral activity of PMF and PMF-OH (**a**), Myricetin rhamnoside (MyrG) and myricetin-3-α-O-ramnosil (1→6)-α-galactoside (MyrGG) (**b**) against HBV *in vitro*. The Ct values obtained for HBV were determined by qPCR. The Ct values for each condition were recorded and quantified based on base to the calibration curve (**c**).

**a)**	HBV											
**Concentration (µM)**	PMF (Ct)			PMF-OH (Ct)			PMF			PMF-OH		
							Median (%)*	SD	N	Median (SD)*	SD	N
**0.0001**	22.65	22.72	22.50	22.38	22.35	22.31	6.67	2.05	3	1.52	0.27	3
**0.001**	23.42	23.50	23.60	22.50	22.36	22.65	51.86	4.19	3	2.50	3.40	3
**0.01**	24.20	24.12	24.30	22.98	22.90	23.11	71.49	3.30	3	29.44	4.08	3
**0.1**	25.82	25.60	25.50	23.60	23.30	23.25	90.22	3.68	3	46.94	3.30	3
**1**	28.42	28.20	28.36	28.75	28.90	28.96	98.69	1.25	3	99.13	0.50	3

*Inhibition percent (%) relative to control

**b)**	HBV											
**Concentration (µM)**	MyrG (Ct)			MyrGG (Ct)			MyrG			MyrGG		
							Median (%)	SD	N	Medina (%)	SD	N
**0.0001**	22.57	22.30	22.21	22.40	22.31	22.12	<0.1	0.00	3	<0.1	0.00	3
**0.001**	22.35	22.30	22.50	22.35	22.54	22.65	<0.1	0.00	3	<0.1	0.00	3
**0.001**	22.79	22.80	22.70	22.30	22.50	22.21	16.10	2.94	3	<0.1	0.00	3
**0.01**	23.40	23.38	23.25	22.45	22.50	22.60	45.61	1.64	3	<0.1	0.00	3
**0.1**	24.40	24.20	24.15	22.50	22.45	22.50	72.36	4.19	3	<0.1	0.00	3
**1**	24.80	24.76	24.79	22.85	22.81	22.91	81.51	3.50	3	21.77	3.24	3
**10**	26.48	26.32	26.20	23.25	23.51	23.46	94.14	2.24	3	48.01	3.30	3
**20**	28.23	28.90	28.40	24.21	24.25	24.18	98.81	0.00	3	71.67	4.24	3

*Inhibition percent (%) relative to control

EXCLI Journal 2019;18:1037-1048 – ISSN 1611-2156
Received: September 20, 2019, accepted: October 29, 2019, published: November 05, 2019

Table 2c: HBV calibration curve. Serial dilutions, from the maximum viral production (100 %), were prepared to construct the calibration curve. Each point corresponds to a percentage of the virus equivalent to 100, 50, 10 and 1 %.

**c**		**HBV**		
**% of viral input**	Viral input (log)	Control (Ct)		
**1**	0	29.00	28.85	28.6
**10**	1	25.27	25.15	25.31
**50**	1.699	23.75	23.9	23.69
**100**	2	22.68	22.3	22.4

Y = -3.076*X + 28.68, R square: 0.98
